# Healthy aging is associated with altered visual gamma band onset and offset responses

**DOI:** 10.1162/imag_a_00401

**Published:** 2024-12-20

**Authors:** Seth D. Springer, Mikki Schantell, Hannah J. Okelberry, Madelyn P. Willett, Hallie J. Johnson, Tony W. Wilson

**Affiliations:** Institute for Human Neuroscience, Boys Town National Research Hospital, Boys Town, NE, United States; College of Medicine, University of Nebraska Medical Center, Omaha, NE, United States; Department of Pharmacology & Neuroscience, Creighton University, Omaha, NE, United States

**Keywords:** oscillation, magnetoencephalography, MEG, gratings, occipital, calcarine

## Abstract

Gamma oscillations have been shown to be critical for basic sensory processing, as well as visual attention and several other higher-order cognitive functions. Aberrant gamma oscillations have also been shown in neuropsychiatric and neurodegenerative diseases. Despite the possible clinical implications of altered gamma activity and emerging stimulation-based interventions targeting gamma, research into age-related changes in gamma oscillatory activity in healthy adults remains sparse. In the current study, we examined the neural oscillations underlying basic visual processing in 87 healthy aging adults using magnetoencephalography (MEG) and a visual grating stimulus. Neural activity elicited by the visual stimulus was imaged using a time-frequency resolved beamformer, and peak voxel time series were computed to characterize the visual oscillatory dynamics underlying these responses. We found significant age-related changes in visual gamma oscillations, but not in visual theta, alpha, or beta oscillations. Specifically, we found age-related increases in gamma band amplitude and inter-trial phase-locking (ITPL) immediately following stimulus presentation (i.e., gamma onset response). Conversely, gamma band amplitude and ITPL following stimulus removal (i.e., gamma offset response) were found to be decreased as a function of healthy aging. Critically, we demonstrated that the decreases in the gamma offset response predicted slower overall processing speed across all participants. Taken together, these findings indicate that healthy aging is uniquely associated with alterations in visual gamma oscillations and that these changes predict participant processing speed.

## 1 Introduction

The neurophysiology underlying cortical visual processing has been extensively studied using a variety of invasive and noninvasive methods and this work has shown that, across a broad range of stimuli, several neural oscillatory responses are reliably detected in the occipital cortices. These include theta responses in the calcarine fissure that occur very early in stimulus processing, exhibit robust developmental changes during childhood (Killanin et al., 2020), and have been widely implicated in the initial encoding of visual stimuli (Fries, 2015; Landau & Fries, 2012). In parallel, robust gamma oscillations also commonly emerge in occipital cortices and these responses have been linked to cortical computation and GABA inhibition (Buzsáki & Wang, 2012; Fries, 2009). Slightly later, there is frequently a strong suppression of alpha and beta band activity in the lateral occipital regions, which has been shown to represent active visual processing in higher-order visual areas (Hanslmayr et al., 2012; Heinrichs-Graham & Wilson, 2015; Neuper & Pfurtscheller, 2001; Proskovec et al., 2016; Wiesman et al., 2017).

Previous work has demonstrated that the sharp increase in neural activity following the appearance of a visual stimulus (i.e., onset response), the weaker sustained activity when the stimulus remains in the visual field (i.e., sustained response), and the activity elicited by the removal of that same stimulus (i.e., offset response) are distinct processes (Di Lollo et al., 2000; Kline & Baffa, 1976; Somervail et al., 2022). The functional relevance of each of these separate neural processes is readily apparent, with navigation through the environment being dependent on the ability to perceive visual stimuli appearing, remaining in view, and disappearing from the visual field. Though studies of the visual onset and sustained responses are quite common, research focusing on the visual offset response is scarce. This discrepancy may be due to the fact that the strength of the offset response is tightly linked to stimulus characteristics such as contrast, spatial frequency, and the total duration for which the stimulus is presented (Török et al., 1992; Vassilev et al., 1983; Wilson, 1983). Thus, the visual offset response is not present in all visual stimulation paradigms. Though visual processing is supported by oscillatory activity across several frequencies (i.e., theta, alpha, beta, and gamma; see above), the only spectral window with onset, sustained, and offset responses to visual stimuli is gamma (Gaetz et al., 2012; Sannita, 2000).

Neural oscillations in the gamma band (~20–100 Hz) have been found in a variety of disparate brain regions (e.g., visual, auditory, motor, parietal (Spooner, Arif, et al., 2021; Van Der Werf et al., 2008; Wiesman & Wilson, 2019b; Wilson et al., 2007, 2008, 2018)) and are associated with many unique cognitive functions (i.e., memory, attention, sensory processing; Fries et al., 2001; Roux et al., 2012; Wiesman et al., 2017). Various early accounts proposed that gamma oscillations mediated the binding of separate stimulus features into a coherent visual precept (i.e., “binding by synchrony”; Gray et al., 1989; Gray & Singer, 1989). However, the broad involvement of gamma band activity across so many regions and during a wide variety of different cognitive functions has led others to suggest that gamma activity serves a less specific, more foundational role in cortical computations (e.g., synchronizing the output of excitatory neurons; Başar-Eroglu et al., 1996; Fries, 2009, 2015; Merker, 2013). In the occipital cortices, gamma oscillations have been shown to be crucial to various aspects of visual processing, including gestalt perception (Keil et al., 1999; Tallon-Baudry & Bertrand, 1999), color processing (Stauch et al., 2022), and the processing of grating stimuli (Muthukumaraswamy & Singh, 2013). Further, these visual gamma responses have been shown to have high test-retest reliability (McCusker et al., 2020; Tan et al., 2016), with amplitude and peak frequency characteristics that are highly dependent on stimulus properties such as contrast and spatial frequency (Bartoli et al., 2019; Hermes et al., 2015; Schadow et al., 2007).

Beyond its role in typical brain functions, gamma band oscillations have been investigated in a variety of neuropsychiatric disorders. Specifically, alterations in gamma band activity have been demonstrated in individuals with schizophrenia (Sun et al., 2013; Tada et al., 2016), autism (An et al., 2018; Wilson et al., 2007), and Alzheimer’s disease (AD; Chan et al., 2022; Wiesman et al., 2021). Further, groundbreaking studies in mouse models of AD have shown that gamma band entrainment, either through direct interneuron stimulation or sensory entrainment, is effective in clearing disease-related pathological proteins (i.e., amyloid beta and hyperphosphorylated tau) while improving cognitive function (Adaikkan & Tsai, 2020; Iaccarino et al., 2016; Martorell et al., 2019). More recently, these findings have been extended to human participants with clinical trials demonstrating that this visual gamma band stimulation may slow the progression of AD-related functional degeneration (Benussi et al., 2021). This is unfortunate, as a more thorough understanding of how healthy aging modulates visual gamma band responses is critical to distinguishing normal changes across the lifespan from those associated with pathological processes.

In this study, we utilized the high spatiotemporal precision of magnetoencephalography (MEG) to investigate age-related alterations in visual cortical oscillations. We were particularly interested in disentangling the unique gamma oscillations in response to visual stimulus appearance (i.e., onset), the stimulus remaining on the screen (i.e., sustained), and the removal of the stimulus (i.e., offset). To elicit these responses, we used a visual grating paradigm that is known to evoke robust gamma oscillations. Based on the few previous studies of visual gamma oscillations in healthy older adults, we hypothesized that there would be significant age-related reductions in gamma band amplitude and inter-trial phase locking across all gamma responses.

## 2 Methods

### 2.1 Participants

Eighty-seven adults with a mean age of 45.84 (SD = 13.20) years and a range of 20.22 to 66.98 years were selected for inclusion in this study. The age range for males was 20.22 to 66.98 years and that for females was 21.42 to 66.89 years. These participants were chosen from a larger-scale study of accelerated aging in people with HIV, with only the cognitively normal HIV-negative participants being included in this investigation of healthy aging (Spooner, Taylor, et al., 2021; Spooner et al., 2023). Of the 87 adults, 93% were right-handed, 77% were male, 93% were not Hispanic, 12.6% were Black/African American, 7.0% were Asian, 74.7% were Caucasian, 4.6% were more than one race, and the remaining 1.2% preferred not to answer. This distribution corresponds closely to the racial demographics of the surrounding region. Notably, there was no effect of age on years of education (*r*_85_ = -.09, *p* = .379) and all included participants scored in the normal range on a neuropsychological testing battery that probed seven cognitive domains (i.e., processing speed, memory, learning, language, executive function, attention, and motor function). The average number of years of education for our sample was 16.00 years (bachelor’s degree), with an SD of 2.332 years, minimum of 12 years (High School diploma), and maximum of 20 years (doctorate or professional degree). Exclusionary criteria included any medical illness affecting CNS function (e.g., HIV/AIDS, Lupus, etc.), any neurological or psychiatric disorder, cognitive impairment, history of head trauma, current substance use disorders, and the MEG laboratory’s standard exclusionary criteria (e.g., ferromagnetic implants). The Institutional Review Board reviewed and approved this investigation. Each participant provided written informed consent following a detailed description of the study.

### 2.2 Experimental paradigm

**Fig. 1.**
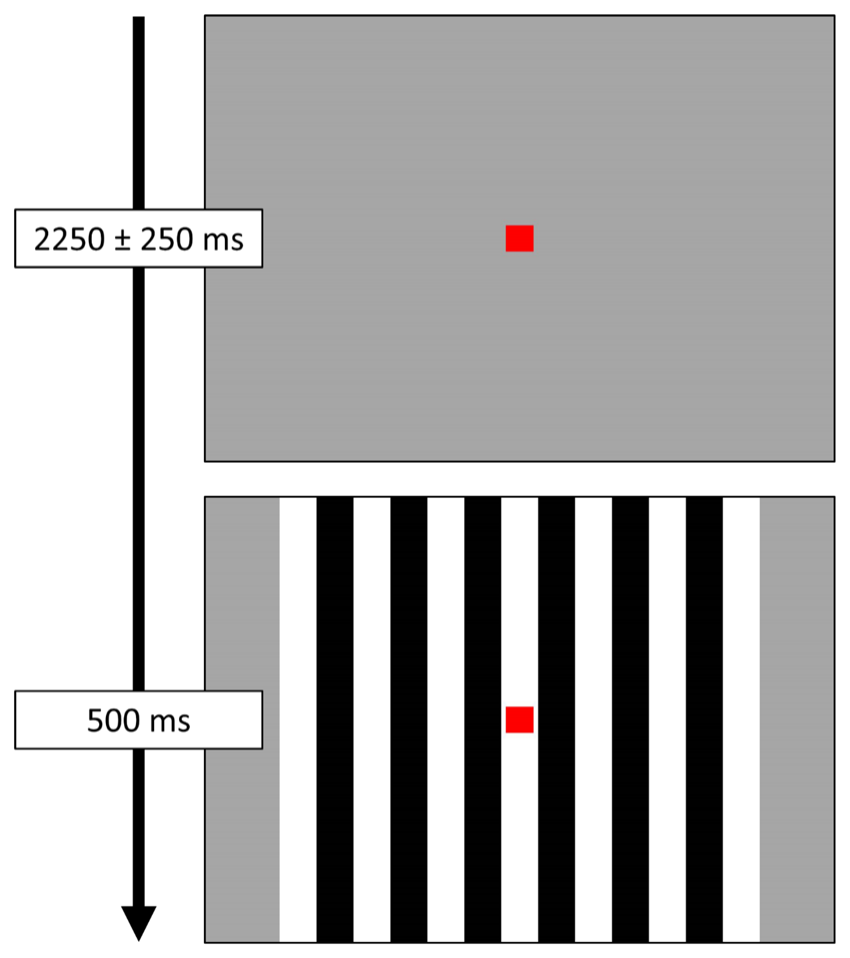
Task paradigm. Participants were instructed to fixate on a red square presented centrally. Following the fixation period, a series of visual gratings appeared around the red square for 500 ms. Each participant completed 120 total trials.

Participants were shown a small, centrally located red fixation square on a grey background. A series of vertical, stationary, square-wave gratings (3 cycles per degree) appeared on the screen for 500 ms, before disappearing and leaving only the fixation square (Fig. 1). The interval between the offset of the grating on one trial and the onset of the grating in the next trial randomly varied between 2000 and 2500 ms. Each participant completed a total of 120 trials, for a total run time of about 5.5 minutes.

### 2.3 MEG data acquisition

All recordings were conducted in a magnetically-shielded room with active shielding engaged. Neuromagnetic responses were sampled continuously at 1 kHz, with an acquisition bandwidth of 0.1–330 Hz, using a MEGIN MEG system with 306 magnetic sensors (MEGIN, Helsinki, Finland). During data acquisition, participants were monitored via real-time audio-visual feeds from inside the shielded room. Subject-wise MEG data were corrected for head motion and subjected to external noise reduction using signal space separation with a temporal extension (Taulu & Simola, 2006).

### 2.4 Structural MRI processing and MEG coregistration

Preceding MEG measurement, four head position indicator (HPI) coils were attached to the participant’s head and localized, together with three fiducial points and at least 100 scalp surface points, with a 3D digitizer (Fastrak, Polhemus Navigator Sciences, Colchester, VT, USA). Once in the MEG, electrical currents with unique frequencies (e.g., 322 Hz) were fed into each of the HPI coils. These HPI coil currents induced measurable magnetic fields, allowing the position of the HPI coils to be tracked relative to the MEG sensors throughout the recording. Since HPI coil locations were also known in head coordinates, all MEG measurements could be transformed into a common coordinate system. With this coordinate system, participant-wise MEG data were coregistered with high-resolution structural T1-weighted MRI data prior to source reconstruction using Brain Electrical Source Analysis (BESA) MRI (Version 2.0, BESA GmbH, Gräfelfing, Germany). Structural MRI data were transformed into standardized space and aligned parallel to the anterior and posterior commissures. Following source analysis, each participant’s MEG functional images were also transformed into standardized space using the same transform and spatially resampled.

### 2.5 MEG preprocessing, time-frequency transformation, and sensor-level statistics

**Fig. 2.**
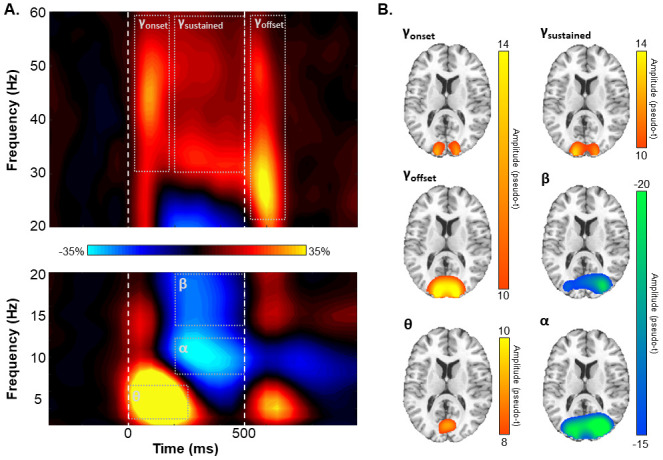
Sensor- and source-level activity. (A) Grand-averaged time-frequency spectrograms from a sensor near parieto-occipital areas (MEG2312), with time (ms) shown on the x-axis and frequency (Hz) on the y-axis. The vertical, dashed white lines denote stimulus onset (0 ms) and offset (500 ms). The colored scale bar between the spectrograms indicates the percentage power change relative to the baseline period (-350 to -50 ms). Significant time-frequency deviations from baseline were identified by cluster-based permutation analysis (see section 2.5) and are highlighted using grey dot boundaries. (B) Grand-averaged beamformer images across all participants for each time-frequency response. Colored scale bars for each beamformer image denote response amplitude in pseudo-*t* units.

Blink, eye movements, and cardiac artifacts were removed from the raw data using signal-space projection, and this correction was accounted for during source analysis (Uusitalo & Ilmoniemi, 1997). The continuous magnetic time series was divided into 1800 ms epochs, with the baseline period being defined as the -350 ms to -50 ms period prior to stimulus presentation (i.e., defined as 0 ms). Subsequently, epochs containing artifacts were removed based on a fixed threshold method, supplemented with visual inspection. Briefly, the amplitude and gradient distributions across all trials were determined per participant, and those trials containing the highest amplitude and/or gradient values relative to this distribution were rejected based on participant-specific thresholds. This approach was employed to minimize the impact of individual differences in sensor proximity to the brain and overall head size, which strongly affect MEG signal amplitude. Importantly, there were no age-related changes in either the amplitude (*r*_85_ = .01, *p* = .923) or gradient (*r*_85_ = .06, *p* = .579) thresholds used for artifact rejection. Artifact-free epochs were then transformed into the time-frequency domain using complex demodulation (Hoechstetter et al., 2004; Kovach & Gander, 2016; Papp & Ktonas, 1977). Lower-frequency responses (i.e., theta, alpha, and beta) were transformed into the time-frequency domain using a resolution of 1 Hz and 50 ms, while higher-frequency responses (i.e., gamma) were transformed using a resolution of 2 Hz and 25 ms. Following time-frequency transformation, spectral power estimates per sensor were averaged across trials to generate plots of mean spectral density per sensor. These sensor-level data were then normalized to the baseline power within each frequency bin, which was calculated as the mean power for that frequency bin during the baseline time period (i.e., -350 to -50 ms; Fig. 2A).

The significant time-frequency windows used for source imaging were determined by statistical analysis of the sensor-level spectrograms across the entire array of gradiometers. Briefly, each pixel was initially evaluated using a mass univariate approach based on the general linear model, followed by cluster-based permutation testing to address the problem of multiple comparisons (Ernst, 2004; Maris & Oostenveld, 2007). Specifically, a two-stage procedure was utilized to minimize false positive results while maintaining sensitivity. The first stage consisted of performing paired-sample t-tests against baseline on each pixel per spectrogram and thresholding the output spectrograms at *p* < .05 to define time-frequency bins containing potentially significant oscillatory deviations from baseline. Bins surviving this threshold (at *p* < .05) were clustered with temporally and/or spectrally neighboring bins that also survived, and cluster values were derived by summing all t-values within each cluster. In stage two, nonparametric permutation testing was used to derive a distribution of cluster-values and the significance level of the cluster(s) from stage one was tested directly using this permuted distribution, which was the result of 10,000 permutations. Based on this permutation analysis, only the time-frequency windows that contained significant oscillatory deviations from baseline at the *p* < .001, corrected, threshold across all participants were subjected to source imaging.

### 2.6 MEG source imaging and statistics

Cortical networks were imaged through a time-frequency resolved extension of the linearly constrained minimum variance (LCMV) vector beamformer (Dalal et al., 2006; Gross et al., 2001; Van Veen et al., 1997). The subject-wise images were derived from the cross-spectral densities of all combinations of MEG gradiometers averaged over the time-frequency range of interest, and the solution of the forward problem for each location on a grid specified by input voxel space. In principle, the beamformer operator generates a spatial filter for each grid point that passes signals without attenuation from the given neural region, while suppressing activity in all other brain areas. The filter properties arise from the forward solution (i.e., lead field matrix) for each location on a volumetric grid specified by input voxel space, and from the MEG data covariance matrix (i.e., cross spectral density matrix). Basically, for each voxel, a set of beamformer weights is determined, which amounts to each MEG sensor being allocated a sensitivity weighting for activity in the particular voxel. Following convention, the source power in these images was normalized per participant using a pre-stimulus period (i.e., baseline) of equal duration and bandwidth (Hillebrand et al., 2005). Such images are typically referred to as pseudo-t maps, with units (pseudo-t) that reflect noise-normalized power differences (i.e., active vs. passive) per voxel. MEG pre-processing and imaging used the BESA software (version 7.1).

After imaging, average whole-brain maps were computed across all participants for the selected time-frequency windows. These 3D maps of brain activity were used to assess the neuroanatomical basis of the significant oscillatory responses identified through the sensor-level analysis. Using these grand averaged (i.e., across all participants) whole-brain maps, we then extracted virtual sensors (i.e., voxel time series) for the peak voxel of each cluster. Specifically, we identified the voxel with the strongest response per hemisphere in the grand average image and computed virtual sensors for that location by applying the sensor weighting matrix derived from the forward solution to the preprocessed signal vector, which yielded a time series for the specific voxel in source space. These virtual sensor time series were then transformed into the time-frequency domain using the same complex demodulation procedure as the sensor-level time-frequency decomposition. From these absolute amplitude time-frequency virtual sensor data, the envelope of spectral power was computed for the frequency range used in each beamforming analysis (i.e., theta: 4–7 Hz, alpha: 8–12 Hz, beta: 14–20 Hz, gamma onset: 30–58 Hz, gamma sustained: 30–58 Hz, and gamma offset: 22–58 Hz). Estimates of the baseline-relative response amplitudes were derived by averaging across the time window used for beamforming in each frequency (i.e., theta: 0–250 ms, alpha: 200–500 ms, beta: 200–500 ms, gamma onset: 25–175 ms, gamma sustained: 200–500 ms, and gamma offset: 525–675 ms) and normalizing these mean values by the mean of the baseline window used in the beamforming analysis of each response. Since we did not have laterality hypotheses, we averaged these data across hemispheres (per response) prior to statistical analyses. Additionally, using the same peak voxel time series data, the envelope of spectral inter-trial phase locking (ITPL) value was computed for the time-frequency ranges used in the beamforming analysis per participant. Specifically, following time-frequency transformation of the single-trial virtual sensor data, ITPL values were calculated across trials for each time-frequency bin within the windows used for beamforming, with the resulting ITPL values ranging from 0 (purely non-phase-locked activity across trials) to 1 (strictly phase-locked activity across trials) per time-frequency bin, and these values were then averaged across the full window used for beamforming for each response (e.g., 0–250 ms, 4–7 Hz for theta (Tallon-Baudry et al., 1996)). These ITPL values were then normalized using the same process as described for the virtual sensor amplitude time series, resulting in baseline-relative ITPL values for each time-frequency window of interest, which were then averaged across hemispheres per response. ITPL values provide information regarding the trial-to-trial consistency of the phase of the response for each participant. Specifically, ITPL tells us how consistently the phases of the neural signal, in a specific time-frequency range and location, are aligning from trial-to-trial for each participant. Finally, to assess whether the age-related alterations in the gamma onset and offset oscillatory responses were driven by phase-locked evoked activity, we computed time-domain averages using the peak voxel time series and probed the classic visual evoked components (i.e., P1, N1, and P2) for aging effects. To reduce the impact of outliers on statistical analyses, participants with values 3.0 SDs above or below their respective group mean were excluded for each analysis.

### 2.7 Statistical analyses and software

All statistical analyses were performed using *JASP* (JASP Team, 2020), and data plots were generated using *ggplot2* (Wickham, 2016). Correlations and multiple regressions were used to model age-related changes in neural activity per response. Single component responses (i.e., theta, alpha, beta, and sustained gamma) were separately correlated with age. Considering the strong relationship between the gamma onset and offset responses (*r*_84_ = .68, *p* < .001), age-related modeling of each of these responses controlled for the other gamma band response. Similarly, gamma onset and offset inter-trial phase locking (ITPL) values were strongly correlated (*r*_85_ = .73, *p* < .001); thus, age-related modeling of the ITPL of each of these responses controlled for the other gamma band ITPL estimate. To investigate the effect of age on the relationship between gamma onset amplitude and gamma offset amplitude, the interaction between age and gamma onset amplitude on gamma offset amplitude was modeled using multiple regression. This interaction was probed using a simple slopes analysis, with the slope values set to the recommended value of ± 1 SD (Aiken & West, 1991). The relationship between gamma onset and offset amplitude and cognitive function was modeled using correlation analyses. Finally, the relationship between age and the classic visual evoked components (i.e., P1, N1, and P2; amplitude and latency) were also modeled using correlation analyses. Considering the relationship between age and the amplitude of the gamma responses, as well as the fact that the neuropsychological domains were corrected for age, the effect of age was removed from the gamma amplitude values in these neurobehavioral models. Finally, the gamma spectral windows differed for the onset (30–58 Hz) and offset (22–58 Hz) responses. To ensure these differences in bandwidth were not driving any of our results, all analyses were recomputed with the shared frequency range (i.e., 30–58 Hz) and none of the significant results changed.

## 3 Results

### 3.1 MEG sensor-level analysis

Sensor-level time-frequency analysis across all participants revealed significant clusters (*p* < .001, corrected) of theta, alpha, beta, and gamma oscillatory activity (Fig. 2A). Theta band activity sharply increased immediately following stimulus presentation and dissipated about 250 ms later (i.e., 4–7 Hz, 0–250 ms; *p* < .001, corrected). Temporally overlapping decreases in alpha (8–12 Hz, 200–500 ms) and beta (14–20 Hz, 200–500 ms) activity began about 200 ms after stimulus onset and lasted until stimulus removal (i.e., 500 ms; both *p*s < .001, corrected). Finally, broadband increases in gamma band activity were observed following stimulus onset (30–58 Hz, 25–175 ms), while the stimulus remained on the screen (30–58 Hz, 200–500 ms), and following stimulus offset (22–58 Hz, 525–675 ms; all *p*s < .001).

### 3.2 Cortical level and time series analysis

To determine the cortical areas generating these significant sensor-level oscillatory responses, we imaged each window using a time- and frequency-resolved beamformer. The resulting whole-brain maps per participant and response were then averaged across all individuals to determine the cortical origins of each oscillatory response (Fig. 2B). Stronger increases in theta activity were observed in the calcarine fissure immediately following stimulus presentation (i.e., 0–250 ms). In contrast, strong decreases in alpha and beta activity were observed from about 200 to 500 ms in the lateral occipital cortices. Finally, bilateral primary visual gamma band activity increased at stimulus onset (i.e., 25–175 ms), was sustained during stimulus processing (200–500 ms), and increased again at stimulus offset (i.e., 525–675 ms).

**Fig. 3.**
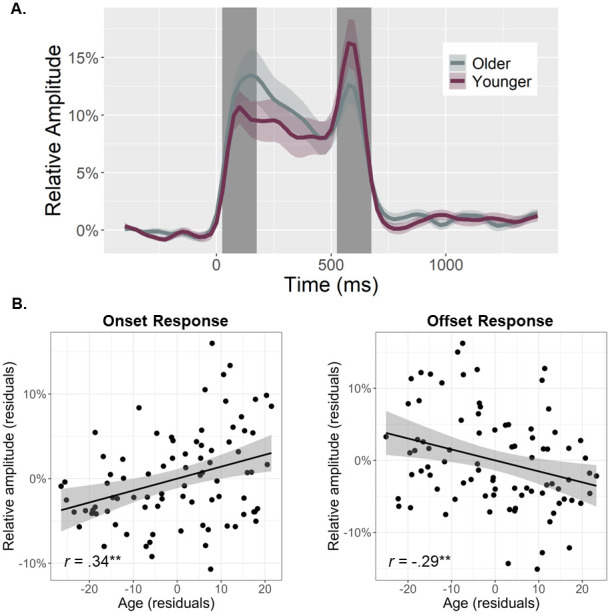
Primary visual gamma amplitude responses and age-related changes in these responses. (A) From the peak voxel showing the strongest gamma neural response, time series were extracted to evaluate changes in response amplitudes as a function of healthy aging during the time windows identified through sensor-level analysis (i.e., onset: 25–175 ms, offset: 525–675 ms; shaded area). The sustained response is not shaded as it was not related to age. Note that all statistics treated age as a continuous variable, but for the sake of visualization, participants have been dichotomized in this figure using a 0.5 SD from the mean cutoff. (B) Scatterplots represent the average onset (left) and offset (right) response amplitude during each respective response time window (i.e., shaded area in A), as a function of age. Lines of linear best-fit and 95% CI (shaded areas in B) are shown. Given the high correlation between onset and offset gamma responses within each participant, the values plotted in (B) are corrected for the other response. **p < .01

**Fig. 4.**
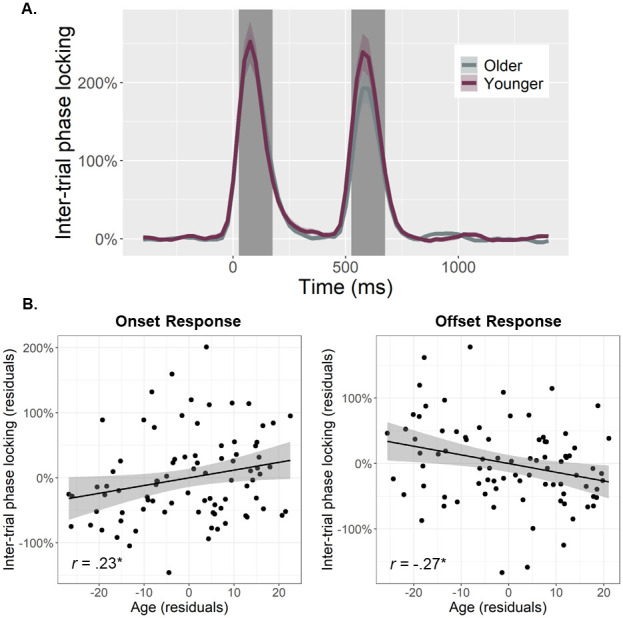
Inter-trial phase locking (ITPL) of visual gamma responses and age-related changes in ITPL. (A) Time series were extracted from the peak voxel based on response amplitude and used to compute ITPL values, which were examined as a function of healthy aging during the time windows identified through the sensor-level analysis (i.e., onset: 25–175 ms, offset: 525–675 ms; shaded area). The sustained response is not shaded as the ITPL value of this response was not related to age. Note that all statistics treated age as a continuous variable, but for the sake of visualization, participants have been dichotomized in this figure using a 0.5 SD from the mean cutoff. (B) Scatterplots represent the average ITPL values for the onset (left) and offset (right) responses during each respective time window (i.e., shaded area in A), as a function of age. Lines of best linear fit and 95% CI (shaded areas in B) are shown. Given the high correlation between onset and offset gamma responses within each participant, the values plotted in (B) are corrected for the other response. *p < .05

To quantify the temporal dynamics of each oscillatory response and evaluate age-related alterations in this neural activity, baseline-relative amplitude and inter-trial phase locking (ITPL) values were computed from virtual sensor time series extracted from the voxel with the greatest amplitude per oscillatory response. Note that the peak voxel was virtually the same (i.e., within one voxel) for all three gamma responses (i.e., onset, sustained, and offset). No age-related amplitude changes were detected for the theta (*r*_84_ = .16, *p* = .131), alpha (*r*_84_ = .02, *p* = .824), beta (*r*_85_ = -.13, *p* = .232), or the sustained gamma response (*r*_84_ = .06, *p* = .560). The same was true for the ITPL measures in the alpha (*r*_84_ = .17, *p* = .128), beta (*r*_84_ = .06, *p* = .588), and sustained gamma (*r*_83_ = -.11, *p* = .336) responses, though there was an age-related increase in theta band ITPL (*r*_83_ = .27, *p* = .012). In contrast, significant age-related *increases* in gamma onset amplitude (*F*_1,82_ = 10.67, *p* = .002; Fig. 3) and ITPL (*F*_1,84_ = 4.02, *p* = .048; Fig. 4), controlling for the same parameters in the gamma offset response (see Methods), were detected in the primary visual cortices. Conversely, significant age-related *decreases* in gamma offset amplitude (*F*_1,82_ = 7.72, *p* = .007; Fig. 3) and ITPL (*F*_1,84_ = 5.43, *p* = .022; Fig. 4), controlling for the onset parameters, were observed in the primary visual cortices. Finally, to assess whether these age-associated changes in gamma onset and offset responses were driven by evoked activity, we computed time-domain averaged activity using the same voxel time series data. We found that the amplitude and latency of the P1 and P2 evoked responses to the onset of the gratings were not related to age, while the N1 amplitude became more negative with increasing age (*p* = .010) and the N1 latency was not related to age (see Supplementary Fig. 1). These three evoked responses were not clearly discernable following the gratings offset. Importantly, inclusion of the amplitude of these components in each of our statistical models did not change any of our findings, which supports the specificity of our age-related findings for gamma oscillatory activity.

**Fig. 5.**
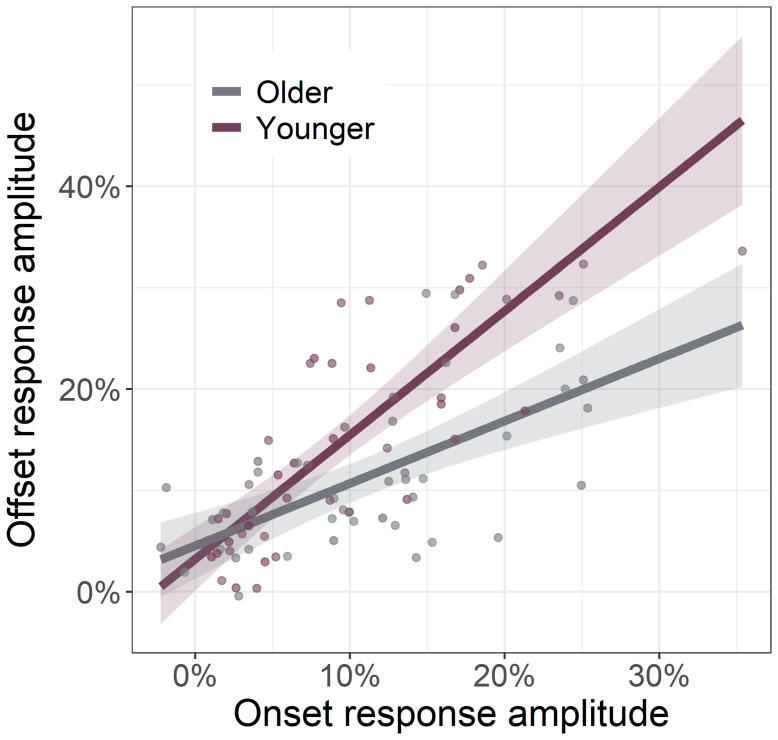
Relationship between gamma onset and offset response amplitude is moderated by age. Across all participants, as the strength of gamma onset response amplitude increased, so too did the strength of the gamma offset response amplitude. This gamma band onset-offset relationship was moderated by age, such that younger individuals had a stronger onset-offset relationship compared to older individuals. Since age was a continuous moderator, the interaction plot is modeled using simple slopes analysis (Aiken & West, 1991), with age set to +1 SD from the mean (59.04 years old; Older) and -1 SD from the mean (32.64 years old; Younger), as is described in the section 2.7. Lines of linear best-fit and 95% CI (i.e., shaded area).

Considering the strong relationship between gamma onset and offset amplitudes (*r*_84_ = .68, *p* < .001), we next investigated if this relationship was altered by age. To this end, we regressed gamma offset amplitude onto gamma onset amplitude with age as a moderator and found a significant interaction between age and gamma offset amplitude (*F*_1,81_ = 9.24, *p* = .003; Fig. 5). Specifically, we found that as participant age increased, the strength of the relationship between gamma onset and offset amplitude decreased. This same age moderation was not found on the relationship between gamma onset and offset ITPL values (*F*_1,83_ = 1.19, *p* = .194).

**Fig. 6.**
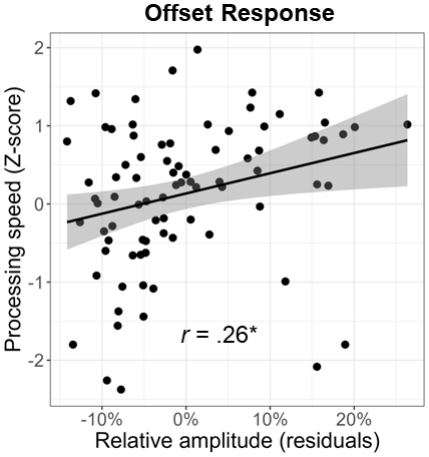
Relationship between processing speed and gamma response amplitude. Across all participants, the strength of the gamma offset response was positively correlated with processing speed. Since the processing speed domain score was age corrected, the effect of age was regressed out of the gamma offset amplitude values prior to conducting this analysis. Line of best linear fit and 95% CI (i.e., shaded area) are shown. *p < .05

Lastly, we investigated the relationship between cognitive performance on the neuropsychological domains and visual gamma amplitude. We found a significant positive relationship between gamma offset amplitude and processing speed (*F*_1,81_ = 6.08, *p* = .016; Fig. 6). This same relationship did not exist between gamma onset amplitude and processing speed (*F*_1,79_ = 2.05, *p* = .157). Note that the processing speed domain score was based on the age-corrected combination of the following tests: Stroop color trials, Stroop word trials, Trail Making Part A, and WAIS-III Digit Symbol Coding. Since all of these scores were age corrected, we regressed out the effect of age from both the gamma onset and offset amplitude values prior to conducting these analyses. Finally, neither gamma onset nor offset responses were significantly related to any of the other neuropsychological domains (*p*s > .05).

## 4 Discussion

Herein, we examined whether healthy aging is associated with altered oscillatory activity serving visual processing using MEG-based source reconstruction and voxel time series analyses. We found no significant age-related changes in occipital theta, alpha, beta, or sustained gamma responses. Our primary results indicated differential alterations in gamma onset and offset responses with healthy aging. Specifically, we found that gamma *onset* amplitude and ITPL increased with increasing age, while gamma *offset* amplitude and ITPL decreased with increasing age. Further, gamma offset amplitude was found to positively predict participant processing speed. Finally, these data demonstrated that age moderates the relationship between gamma onset and offset amplitude. Specifically, younger adults exhibited a strong positive relationship between gamma onset/offset amplitude, which was significantly diminished in older adults. Below, we explore the implications of these novel findings for understanding the impact of healthy aging on visual processing.

The observed responses in the occipital cortices were in broad agreement with previous research on the oscillatory activity underlying visual processing. The theta activity observed at stimulus onset in the calcarine fissure is commonly elicited in studies of visually evoked potentials and has been widely implicated in the initial encoding of visual stimuli (Fries, 2015; Landau & Fries, 2012). The suppression of alpha and beta band activity in the lateral occipital regions has been shown to represent active visual processing in higher-order visual areas (Hanslmayr et al., 2012; Heinrichs-Graham & Wilson, 2015; Neuper & Pfurtscheller, 2001; Proskovec et al., 2016; Wiesman et al., 2017). We found no age-related alterations in occipital theta, alpha, or beta band oscillations, with the exception of an age-related increase in the ITPL value of the theta response. This suggests that the phase of the theta response is more consistent per unit time in older compared to younger participants. The significance of this finding for visual processes is not entirely clear and should be a focus of future work. As has been previously shown, visual gamma band activity can be subdivided into several different types of responses. The visual gamma onset and offset responses are sharp transient increases that coincide with the appearance and disappearance of a visual stimulus, while the sustained gamma response, between onset and offset, is typically weaker than the onset and offset responses but still much stronger than gamma activity during the baseline (Başar-Eroglu et al., 1996; Gaetz et al., 2012; Koelewijn et al., 2011; Murty et al., 2020; Sannita et al., 2001; Schadow et al., 2007; Swettenham et al., 2009). One exception to this can be seen in visual entrainment tasks, where the neural response is tightly locked to the oscillating stimulus through both the onset and sustained aspects (Heinrichs-Graham et al., 2017; Springer et al., 2023; Springer et al., 2022; Wiesman et al., 2019; Wiesman & Wilson, 2019a). Previous work by Muthukumaraswamy et al. (2010) demonstrated that the onset visual gamma response was highly consistent across recording sessions, while the more sustained aspect showed large inter-individual variability in gamma band peak frequency, bandwidth, and amplitude. Herein, source imaging demonstrated that the cortical distribution of both the visual onset and offset responses was virtually identical, with both responses having the same peak in each hemisphere. This is in agreement with previous analyses showing that the onset and offset responses had similar scalp distributions (Parker et al., 1982; Somervail et al., 2022), which we further extend here by demonstrating that the sustained gamma response also originates from the same occipital cortical location as the gamma onset and offset responses.

Our most striking findings were the age-related increases in the visual gamma onset response and decreases in the subsequent offset response, with no age-related changes in the sustained gamma oscillatory response. Interestingly, Murty et al. (2020) found significant decreases in sustained gamma activity with increasing age in a sample that included participants up to 88 years of age. Thus, our negative finding for the sustained gamma response could reflect that our oldest participants were only about 67 years-old. These age-related changes in gamma band amplitudes were mirrored by similar changes in ITPL, with there being stronger phase-locking to stimulus onset and weaker phase-locking to stimulus offset as a function of age. Further, we found that gamma onset and offset response amplitudes were positively correlated with one another, but that this positive relationship became weaker as a function of age. This onset-offset relationship presumably reflects the fact that stimuli which elicit stronger onset responses require stronger offset responses to “clear” the visual cortex once the visual stimulus has been removed. This hypothesis is indirectly supported by previous research which has demonstrated that onset and offset response amplitudes both scale with changes in the same visual stimulus properties (e.g., stimulus size, luminance, spatial frequency; Fortune et al., 2009; Parker et al., 1982). The visual gamma onset response has been associated with the initial encoding of visual stimuli (Busch et al., 2004; Edden et al., 2009; Hoogenboom et al., 2006; Muthukumaraswamy & Singh, 2013); thus, our findings of age-related increases in this response likely indicate more effortful early processing of visual information. Interpretation of the weaker offset response with increasing age is more difficult considering the lack of previous research on visual offset responses. However, we believe that the decrease in offset response amplitude may relate to the observation that older individuals have longer visual persistence (Gaál et al., 2017; Kaur et al., 2020; Kline & Orme-Rogers, 1978; Mewborn et al., 2015).

Visual persistence is the phenomenon in which an individual briefly continues to perceive a visual stimulus after that stimulus has been removed from the visual field. Longer visual persistence leads to slower overall sensory processing, as it takes these individuals longer to “recover” from one stimulus and to prepare to encode the next one (Di Lollo et al., 1982; Kline & Nestor, 1977; Mewborn et al., 2015). Supporting our hypothesis that the gamma offset response is related to visual persistence and thus sensory processing speed, we found a strong positive association between the amplitude of the gamma offset response (during the MEG visual processing task) and processing speed (measured outside of the scanner using neuropsychological assessments). This brain-behavior relationship was specific to the offset response, with there being no relationship between the onset response and processing speed. Similarly, it has been suggested that the longer visual persistence in older individuals may be related to deficits in inhibitory processing (Gaál et al., 2017). This aligns with our findings of age-related decreases in gamma offset activity, as the generation of gamma oscillations has been repeatedly linked to the activity of GABAergic inhibitory interneurons (Brunel & Wang, 2003; Buzsáki & Wang, 2012), but is less consistent with our negative findings for the sustained gamma response. Overall, our findings indicate that visual gamma oscillations, particularly the offset response, are useful metrics for probing age-related changes in visual processing and how these changes affect cognitive function.

Before closing, it is important to acknowledge several limitations of the current work. First, we used a spatial gratings stimulus; while this is one of the most widely used paradigms in basic visual research, our age-related findings may not generalize to all visual input. Second, as noted above, our oldest participant was about 67 years old and further research will be critical to investigate how the current findings may change as older individuals are included. Another limitation is the cross-sectional nature of our study and future work in this area should consider longitudinal designs. Thus, we are unable to directly relate within-subject changes in age to alterations in gamma oscillatory activity. Finally, future studies should integrate eye-tracking to precisely monitor for eye movements. This would enhance experimental rigor and enable trials where participants were not closely attending to the stimulus to be excluded. Nonetheless, taken together, we found that gamma band cortical activity increased following both the appearance of a sinusoidal grating stimulus (i.e., onset) and when that stimulus was removed (i.e., offset). However, the amplitude and ITPL of these gamma band oscillations were differentially related to healthy aging, despite the fact that they originated from the same cortical locations. Specifically, older individuals had stronger gamma band visual onset responses, along with weaker visual offset responses. Critically, these age-related decreases in the visual offset response predicted worse processing speed across all participants. Gaining a deeper understanding of these age-related changes in basic visual processing could hold major implications for designing specific interventions aimed at mitigating these visual changes, thus leading to better cognitive outcomes in older individuals.

## Supplementary Material

Supplementary Material

## Data Availability

The data and code that support the findings of this study are available from the corresponding author, Dr. Tony W. Wilson, upon reasonable request.
