# Supplementary material for: Healthy aging is associated with altered visual gamma band onset and offset responses
Source: Imaging Neurosci (Camb). Author manuscript; Available in PMC 2025 Mar 6. (PMC11873763; doi:10.1162/imag_a_00401)
Supplement: Supplemental Material [file NIHMS2044791-supplement-Supplemental_Material.pdf]

Manuscript Title: Healthy Aging is Associated with Altered Visual Gamma Band Onset and Offset Responses

Authors: Springer SD, Schantell M, Okelberry HJ, Willett MP, Johnson HJ, Wilson TW

Supplementary Material

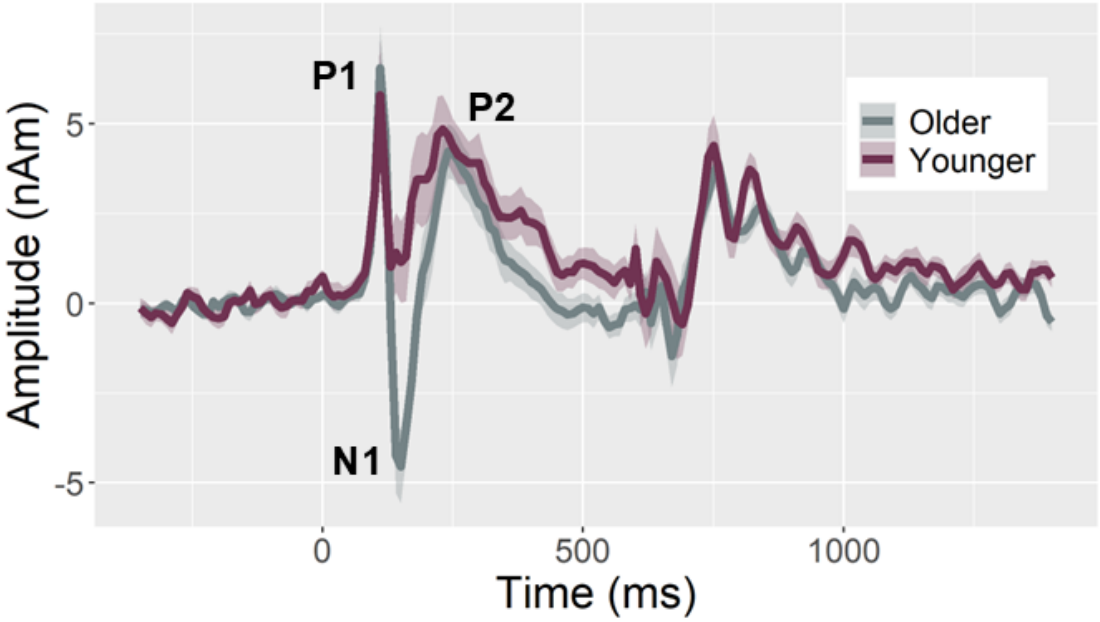

Supplementary Figure 1. Primary visual time-domain average response. From the peak voxel showing the strongest gamma neural response, time series were extracted and averaged across trials to evaluate changes in response amplitude values and latencies as a function of healthy aging. Note that all statistics treated age as a continuous variable, but for the sake of visualization, participants have been dichotomized in this figure using a 0.5 SD from the mean cutoff.
